# Specificity Protein 1 Transcription Factor Regulates Human ARTS Promoter Activity through Multiple Binding Sites

**DOI:** 10.1371/journal.pone.0120072

**Published:** 2015-03-19

**Authors:** Feifan Xu, Wei Sun, Pan Li, Jinling Chen, Dandan Zhu, Xiaolei Sun, Jianxin Wang, Jinrong Feng, Ke Song, Yinong Duan

**Affiliations:** 1 Department of Pathogen Biology, School of Medicine, Nantong University, 19 Qixiu Road, Nantong 226001, Jiangsu, People’s Republic of China; 2 Clinical Laboratory, The Sixth People’s Hospital of Nantong, Nantong 226001, Jiangsu, People’s Republic of China; 3 Laboratory Medicine Center, Affiliated Hospital of Nantong University, Nantong 226001, Jiangsu, People’s Republic of China; University of Saarland Medical School, GERMANY

## Abstract

Apoptosis-related protein in the TGF-β signaling pathway (ARTS) is an unusual mitochondrial Septin-like protein which functions as a tumor suppressor. There are various splice variants derived from the human Septin4 gene, one of which is ARTS, also known as Septin4_i2. Unlike other Septin4 members, ARTS can induce apoptosis in many cells, however, the underlying molecular mechanism for the transcriptional regulation of ARTS has yet to be deciphered. In this study, we attempted to analyze the promoter region of ARTS in cultured HEK-293T and LX-2 cells with the purpose of elucidating the underlying transcriptional mechanisms driving ARTS expression. We effectively demonstrated that the -824 to -5 bp region of the ARTS promoter was essential for ARTS transcription and identified four putative specificity protein 1 (Sp1) binding sites within this core promoter region. ChIP analysis showed that Sp1 protein could bind to two of these sites (-735/-718 and -173/-157) and mutation of each Sp1 binding site led to a significant decrease in ARTS promoter activity. In conclusion, all the results indicated that the Sp1 transcription factor could contribute to ARTS gene transcription. The underlying molecular events of the specific promoter of ARTS could also be used to explain why ARTS is selectively silenced during some human diseases. This would provide basis for further study on the function of ARTS on cell apoptosis.

## Introduction

Septins are a family of GTP-binding proteins, which were initially discovered in yeast as proteins associated with cell division. They were subsequently found to exist in fungi, algae, insects, and vertebrates [1,2] and often play an important role in cytokinesis, exocytosis, vesicle trafficking, membrane dynamics, tumorigenesis, apoptosis and DNA repair [3–5]. To date, at least 14 human Septin (Sept) genes (Sept1 to Sept14) which encode dozens of different protein isoforms have been identified [6]. Recent researches have implicated the involvement of Septins in various human diseases, including neurological disorders, infections, and a variety of cancers such as myeloid neoplasia and colorectal cancer [7–9]. One member of the human Septin family, Sept4, has been reported to play a role in Parkinson’s disease, acute lymphoblastic leukemia (ALL) and spermatogenesis [10–12].

Sept4_i1 and Sept4_i2 are two of the six known splice variants which are encoded by the human Sept4 gene. Sept4_i2, also known as ARTS (Apoptosis-related protein in the TGF-β signaling pathway), is an unusual mitochondrial Septin-like protein which exhibits pro-apoptotic activity by binding to inhibitors of apoptosis proteins (IAPs) [13,14]. Edison et al. reported [15] that ARTS can activate the Bid protein by binding to X-linked IAP (XIAP) to initiate caspase activation, resulting in mitochondrial outer membrane permeabilization (MOMP) and amplification of cell apoptosis. Many pro-apoptotic stimuli, such as the transforming growth factor-β1 (TGF-β1), Fas, etoposide, staurosporine and arabinoside, are reported to have the ability to induce apoptosis trough ARTS/XIAP binding [11,16]. These stimuli can increase the expression level of ARTS by inhibiting the process of ubiquitination-induced degradation [16,17]. Significantly, ARTS expression is frequently lost in some diseases, such as in ALL, where it functions as a tumor suppressor and is selectively lost within the majority of patients [11]. ARTS expression is also reduced in the brains of schizophrenic patients and involved in the pathogenesis of the disease [18]. In Parkinson’s disease, Parkin can bind to ARTS and thus restrict the levels of ARTS through the Ubiquitin Proteasome System (UPS)-mediated degradation [19].

Similar with other Septin proteins, ARTS contains a conserved central GTPase domain flanked by variable N- and C-termini, with proline-rich N-terminal and coiled coil C-terminal domains [14]. Importantly, ARTS contains a 27-residue domain which is essential for the binding of ARTS to XIAP at the C-terminal and lacks an N-terminal 20-residue domain which was found in Sept4_i1 [14,20]. In addition, the P-loop domain (GES-GLGKST), which is also conserved among Sept4 members, is reported to mediate ARTS-induced apoptosis [13,21] however, in Sept4_i1, the P-loop domain cannot mediate the same pro-apoptotic function [13,16]. There are two major distinct promoters in Sept4: a distal promoter that generates ARTS and a proximal promoter that produces Sept4_i1. These two different promoters may be one of the reasons responsible for the differences in structural and functional features between ARTS and Sept4_i1 [14]. Hence, in this study, we attempted to analyze the promoter regions of ARTS in cultured HEK-293T and LX-2 cells with the goal of elucidating the underlying transcriptional mechanisms driving ARTS expression.

## Materials and Methods

### Cell culture

HEK-293T cells (a human embryonic kidney cell line [22]) were purchased from Cell Bank of Typical Culture Preservation Commission (Chinese Academy of Sciences) and LX-2 cells (a human hepatic stellate cell line [23]) were preserved in our laboratory [24]. All the cells were maintained in Dulbecco’s Modified Eagle’s Medium (DMEM, Gibco, USA), supplemented with 10% fetal bovine serum (FBS, Hyclone, USA), 100 units/ml of penicillin, and 100 μg/ml streptomycin solution, at 37°C in a humidified incubator with 5% CO_2_.

### Bioinformatics analysis of the promoter region

The 2.3 kb sequence of the ARTS promoter (-2045 bp to +263 bp), containing the ATG start site, was obtained from the GeneBank nucleic acid sequence database of the National Center for Biotechnology Information (NCBI, http://www.ncbi.nlm.nih.gov/). Transcription factor binding sites were identified using the network tool TFSEARCH (http://www.cbrc.jp/research/db/TFSEARCH.html) and the MatInspector software tool of Genomatix.

### Luciferase reporter constructs

Polymerase chain reaction (PCR) primers (S1 Table) were used to amplify the promoter fragment (-2045 bp to +263 bp) of the human ARTS from genomic DNA of LX-2 cells. The fragment was then cloned into the *Kpn* I and *Sac* I restriction sites of the pGL3-Basic vector (Promega, USA) and named as pGL3-A1 plasmid. A series of deletion constructs of the ARTS promoter was generated by PCR amplification from the cloned pGL3-A1 plasmid (-2045 bp to +263 bp), and named as pGL3-A2, pGL3-A3, pGL3-A4 and pGL3-A5, respectively. All the primers are shown in S1 Table with the restriction sites underlined.

Within the pGL3-A4 deletion constructs, the putative specificity protein 1 (Sp1) binding sites in the human ARTS promoter were mutated by overlap extension PCR, according to the method of Ho et al. [25]. The primers used to introduce these mutations are listed in S1 Table, with the mutated residues underlined. All mutations were cloned into the *Kpn* I/*Sac* I sites of the pGL3-Basic vector. All constructs, both deletion and point mutations, were verified by DNA sequencing.

### Transient transfection and luciferase reporter assay

For the luciferase reporter assay, LX-2 and HEK-293T cells were transiently transfected with reporter plasmids using FuGENE Transfection Reagent (Promega, USA) according to the manufacturer’s instructions. Briefly, cells were seeded in a 24-well plate at 5×10^4^ cells/well in medium without antibiotic/antimycotic, and incubated to 70–80% confluency. Before transfection, 1.2 μl of FuGENE reagent was diluted with 25 μl of DMEM and incubated at room temperature for 5 min and then 0.4 μg of reporter plasmids was added to the FuGENE/medium mixture and incubated for another 15 min at room temperature. The FuGENE/medium/DNA mixture was then added to 500 μl cell culture media. After transfection, the cells were cultured for 48 h and harvested for luciferase activity analysis using the Dual-Luciferase Reporter Assay system (Promega, USA) according to the manufacturer’s instructions. Luciferase activity analysis was then performed on a luminometer (Promega, USA). The reporter plasmids which were cloned into the pGL3-Basic vector could express firefly luciferase. The pRL-TK plasmid (Promega, USA) expressing renilla luciferase was used as an internal control.

### Western blot

Proteins were extracted from lysates of LX-2 and HEK-293T cells using RIPA buffer (Beyotime, China) and subjected to 10% sodium dodecyl sulfate-polyacrylamide gel electrophoresis (SDS-PAGE). The proteins were then electro-transferred from the gels onto polyvinylidene fluoride (PVDF) membranes, followed by blockage with 5% nonfat milk. Then the PVDF membranes were incubated with either anti-ARTS antibody (Prosci, USA) or anti-glyceraldehyde phosphate dehydrogenase (GAPDH) antibody (Goodhere, China), followed by incubation with the corresponding secondary antibodies (Santa Cruz, USA). The membranes were then visualized using an enhanced chemiluminescence kit (Millipore, USA).

### Chromatin immunoprecipitation assay (ChIP)

ChIP was performed by using the ChIP Kit from Thermo (USA) according to the manufacturer’s protocol. HEK-293T cells were cross linked in 1% formaldehyde and then digested with Micrococcal Nuclease (MNase). Anti-Sp1 antibody (Santa Cruz Biotechnology, sc-59x) was added to precipitate DNA-protein complexes and normal rabbit IgG was used as a negative control. Standard PCR was used to detect Sp1-binding sequences with ChIP purified DNA as the template. PCR primers are listed in S1 Table and the PCR conditions were as follows using DNA polymerase (TransGen Biotech, China): 95°C for 2 min, 33 cycles of 95°C for 20 s, 57°C for 20 s and 72°C for 30 s, with a final extension of 72°C for 5 min.

### Statistical analysis

Results were presented as mean±SD of at least three independent experiments, which were performed in triplicate. Data were analyzed by the one-way ANOVA method using SPSS 15.0. Statistical significance was defined as *P*<0.05.

## Results

### Identification of the putative ARTS promoter

**Fig 1:**
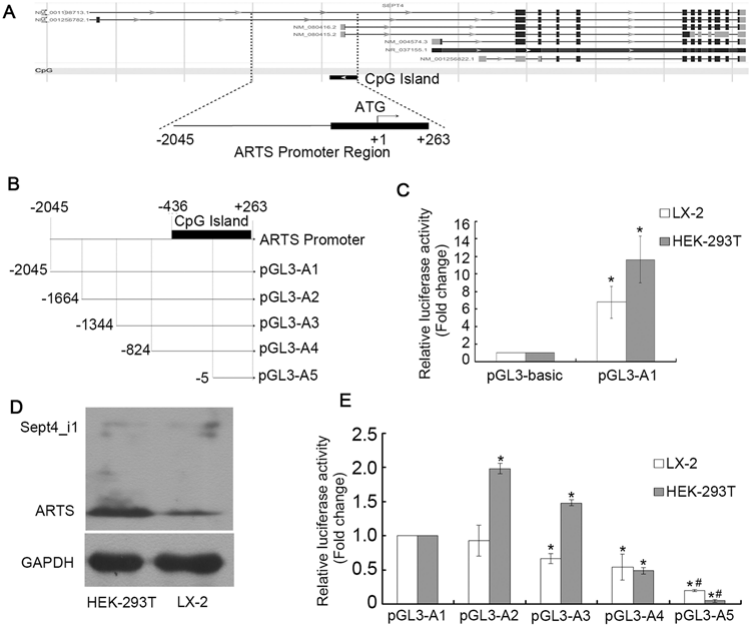
Identification of the putative ARTS promoter. (A) Schematic representation of the human Sept4 gene features. The distinct transcript variants of human Sept4 gene were obtained and analyzed using NCBI Epigenomics. The structure of the putative ARTS promoter region (from -2045 bp to +263 bp), containing a CpG island. (B) Schematic representation of the ARTS promoter deletion constructs generated in the pGL3-basic vector. (C) The relative luciferase activity of pGL3-A1 in HEK-393T and LX-2 cell lines was detected by luciferase reporter assay. *P<0.05, as compared with the relative luciferase activity of pGL3-basic vector. (D) The endogenous expression of ARTS (32 kDa) in HEK-393T and LX-2 cell lines was detected by western blot. (E) The relative luciferase activities of the deletion constructs in both HEK-393T and LX-2 cell lines were observed by luciferase reporter assay. *P<0.05, as compared with the relative luciferase activity of pGL3-A1 in each cell line. ^#^ *P*<0.05, as compared with the relative luciferase activity of pGL3-A4 in each cell line.

ARTS is a major splice variant of the Sept4 gene with a putative promoter located at the position from -2045 bp to +263 bp, and according to NCBI Epigenomics (http://www.ncbi.nlm.nih.gov/epigenomics/), a CpG island is present in the promoter region. We therefore focused on the 2.3 kb genomic region (-2045 bp to +263 bp) containing the ARTS translation initiation codon, ATG (+1 bp to +3 bp, Fig. 1A). No TATA and CAAT elements were identified in the region.

The entire 2.3 kb sequence was cloned into the pGL3-basic vector to generate the pGL3-A1 plasmid (Fig. 1B). The pGL3-A1 plasmid was transiently transfected into LX-2 and HEK-293T cell lines to investigate whether the putative promoter region was able to drive the expression of the luciferase reporter gene carried in pGL3-basic vector. As shown in Fig. 1C, this pGL3-A1 plasmid which contained the putative promoter region could drive the reporter gene expression successfully, as compared to the pGL3-basic vector. The levels of promoter activity differed in the two cell lines, with a 6.76-fold increase in activity in the LX-2 cells and a stronger, 11.65-fold increase in the HEK-293T cells. This difference in promoter activity between the two cells lines was consistent with protein expression levels, as evidenced by western blot analysis (Fig. 1D).

In order to define the sequence responsible for ARTS basal transcription, we generated a series of deletion constructs according to the presence of the predicted transcription factor binding sites. The deletion constructs are schematically represented in Fig. 1B (pGL3-A2, pGL3-A3, pGL3-A4 and pGL3-A5 plasmids). All of the plasmids were transiently transfected into both cell lines and the activities of the promoters for each deletion construct were measured (Fig. 1E). In LX-2 cells, the promoter activities of the deletion plasmids were all decreased, when compared to the luciferase activity of the pGL3-A1 construct (*P*<0.05). Deletion of the region from position -824 bp to -5 bp resulted in a significant decrease in both the promoter activity and the luciferase activity of pGL3-A5, compared to the activity of the pGL3-A4 construct (*P*<0.05). Similar results were also observed in HEK-293T cells. All results indicated that the region between -824 bp to -5 bp of the promoter was critical for the expression of the ARTS gene, suggesting that this region might contain some functional elements.

### Identification of putative transcription factor binding sites in the -824 to -5 bp region of the ARTS promoter

**Fig 2:**
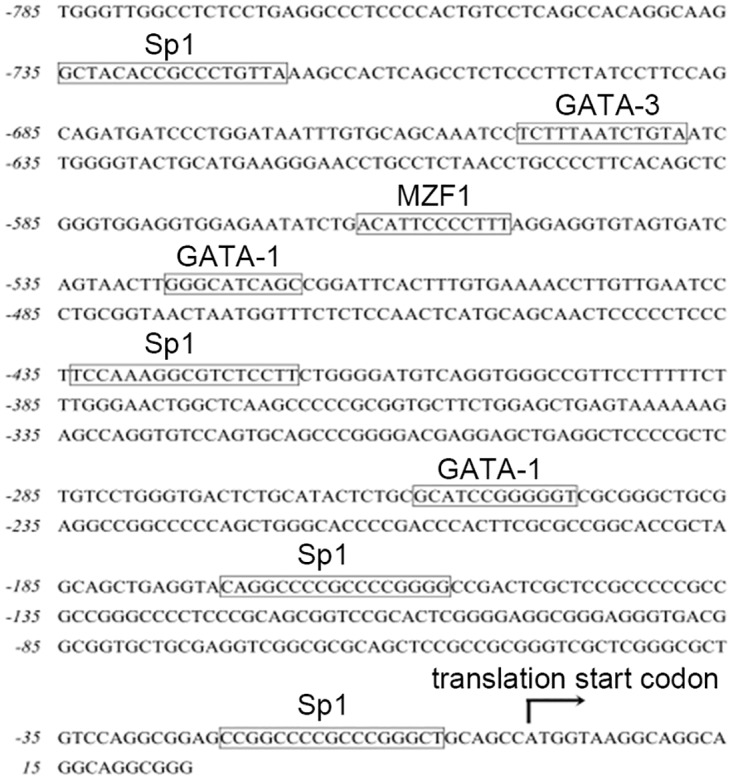
Putative transcription factor binding sites in ARTS promoter. The binding sites in the region from -824 to -5 bp of the promoter were screened using TFSEARCH and MatInspector. Boxed sequences indicate the predicted sites.

The results above revealed that the region from -824 bp to -5 bp is responsible for the expression of the ARTS gene. We thus sought to identify transcription factors which could be involved in this regulation. By using the MatInspector and TFSEARCH tool, we found numerous putative transcription factor binding sites in the region from -824 bp to -5 bp, including binding sites for Sp1, GATA-1, MZF1 as well as other transcription factors (Fig. 2). It is known that Sp1 plays a critical role in the basal level of transcription in the majority of promoters containing Sp1-binding sites [26,27] and that it is able to activate transcription initiation through recruiting the TATA binding protein-transcription factor IID (TBP-TFIID) complex in TATA-less promoter regions [28]. Hence, we hypothesized that Sp1 binding sites might contribute to the regulation of ARTS expression and we focused our further study on this topic.

### Sp1 binds to the ARTS promoter in vivo

**Fig 3:**
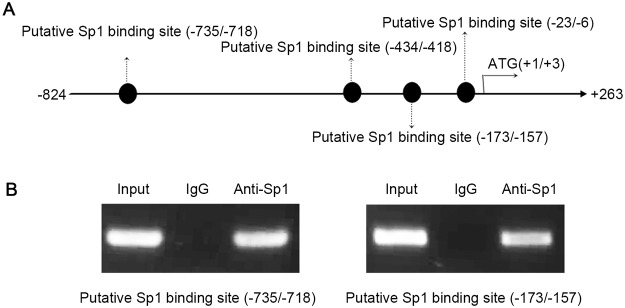
Sp1 protein binds to Sp1 binding sites in ARTS promoter. (A) The schematic representation of the position of four putative Sp1 binding sites in the human ARTS promoter region (-824 bp to -5 bp). (B) The specific binding of Sp1 protein to the putative Sp1 binding sites in the ARTS promoter region was analyzed by ChIP. Protein/DNA complexes from the sonicated lysates of HEK-293T cells were immunoprecipitated with anti-Sp1 antibody with rabbit IgG used as a control. The results showed that the expression of Sp1 could be detected at the putative binding sites of -735/-718 and -173/-157, but not -434/-418 and -23/-6.

To determine whether Sp1 could bind to these four putative Sp1 binding sites, we performed ChIP assays on chromatin obtained from HEK-293T cells precipitated with anti-Sp1 antibody. The predicted Sp1 binding sites in the ARTS promoter are shown in Fig. 3A. The expression of Sp1 could be detected at the binding sites at -735/-718 and -173/-157, as shown in Fig. 3B. Rabbit IgG was used as negative control, and no PCR amplification products were observed. However, the putative Sp1 binding sequences (-434/-418 and -23/-6) failed to be immunoprecipitated with the anti-Sp1 antibody. The results of these ChIP assays revealed that Sp1 protein could bind to the ARTS promoter in live cells via Sp1 binding sites at -735/-718 and -173/-157.

### Sp1 binding sites located in the ARTS promoter are required for its activity

**Fig 4:**
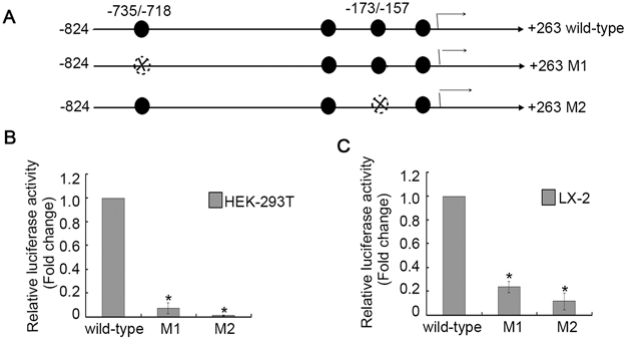
Sp1 binding sites are required for ARTS promoter activation. (A) The schematic representation of the mutation project. The constructs were then transiently transfected into HEK293 cells (B) and LX-2 cells (C) and promoter activities were determined by luciferase assay. The activities for the promoter of M1 and M2 plasmids were reduced in both cell lines, compared with the activity of the wild-type plasmid (*P<0.05).

To observe the function of Sp1 in the modulation of ARTS transcription activity, site-directed mutations within the core sequence of each Sp1 site were performed, as displayed in Fig. 4A. Luciferase assays were then conducted and the luciferase activity of each mutation construct was compared with the associated wild-type construct in both LX-2 and HEK-293T cells. As evidenced in Fig. 4B and C, the luciferase activities of the Sp1 single-site mutant plasmids, M1 and M2, were significantly reduced in both cell lines compared to respective wild-type construct. These results strongly supported the hypothesis that Sp1 could positively drive ARTS gene transcription through Sp1-binding sites. Together with site-directed mutagenesis and binding analysis data, we concluded that Sp1 could contribute to the modulation of the ARTS promoter activity.

## Discussion

Previous studies have showed that Sept4 is a tissue- and cell-type-specific gene expressed in colorectal cancer and malignant melanoma, but it is also expressed at low or undetectable levels in other diseases like pulmonary adenocarcinoma and acute lymphoblastic leukemia [11,29]. In our study, we found that a CpG island (-436 bp to +374 bp) overlapped in the ARTS promoter. Since DNA methylation of the gene promoter was known to be associated with the regulation of tissue- and cell type-specific gene expression [30,31], we speculated that the methylation of the CpG island was responsible for the difference in expression of Sept4 in different cells and tissues. This theory was proved by Elhasid et al. [11], who demonstrated that epigenetic silencing of ARTS by methylation is one mechanism of losing ARTS expression, and that a lack of ARTS expression in cancer cells is responsible for inhibition of apoptosis in malignant leukemic blasts. Thus, CpG methylation may serve as a modulator of transcription to regulate ARTS expression in a cell- and tissue-specific fashion. Interestingly, apoptosis, another characteristic of ARTS expression and function, could be induced by ARTS, but not other members of Sept4 family. However, the potential mechanism for this remains unclear. Since the different promoters of ARTS and Sept4_i1 may contribute to the different structural and functional features [14], we directed our focus on the underlying molecular mechanisms by which ARTS transcription could be derived.

For the promoter characterization, we identified that the plasmid carrying the putative promoter of ARTS (-2045 bp to +263 bp) could drive reporter gene expression in both the HEK-293T and LX-2 cell lines. Bioinformatics analysis revealed that several transcription factor binding sites and a CpG island were identified in the promoter region of ARTS gene, but no functional TATA or CAAT elements were present. A series of 5′-deleted fragments of the ARTS promoter were designed based on the presence of the transcription factor binding sites. The results suggested that the upstream region of the ARTS initiation codon from -824 bp to -5 bp, containing all four Sp1 binding sites, was essential for the transcription of the ARTS gene. However, the promoter activities of pGL3-A2 and pGL3-A3 plasmids were significantly increased in HEK-293T cells, compared with those in pGL3-A1. We postulated that some regulatory elements might exist in the deletion region from position -2045 bp to -1344 bp to negatively regulate the expression of ARTS promoter activity in HEK-293T cells, but not in LX-2 cells. The ARTS promoter (pGL3-A2 and pGL3-A3) activity could be up-regulated in HEK-293T cells when these possible regulatory elements were deleted. This phenomenon was also reported in the promoter of human glycoprotein VI gene [32].

Sp1 is a zinc finger transcription factor that binds to the GC-rich motifs of many promoters. Previous studies have reported that Sp1 regulates the expression of TATA-less genes containing Sp1-binding sites within the promoter [26,27]. Sp1 protein may bind TATA-less and/or GC-rich promoter regions to recruit TATA-binding protein-associated factors, then interact with TFIID to stimulate transcription initiation [28,33,34]. In our study, we have demonstrated that Sp1 could interact with the ARTS promoter in HEK-293T cells. Furthermore, Sp1 only bound to two Sp1 binding sites (-735/-718 and -173/-157) in ARTS promoter, but not the other two binding sites (-434/-418 and -23/-6). We incorporated site-directed mutagenesis into the Sp1 binding sites in ARTS promoter to explore whether the predicted Sp1 factor binding sites are functionally relevant. Mutation of each Sp1 binding site (-735/-718 and -173/-157) could induce a significant decrease in promoter activity, compared with the wild-type construct. As a whole, the results indicated that Sp1 played a critical role in ARTS promoter transactivation.

In conclusion, our study identified the critical region in the ARTS promoter. We have also demonstrated that the Sp1 transcription factor could regulate the activity of the ARTS promoter through multiple Sp1 binding sites. We intend to focus our future research on characterization of the Sp1 protein functions in apoptosis of LX-2 cells through ARTS associated pathways, which provides a basis for study on the function of ARTS in hepatic fibrosis.

## Supporting Information

S1 Table Primers used in the study. All the primers used in this study are shown and the restriction sites or the mutated residues are underlined.
(PDF)
