# Supplementary material for: Specificity Protein 1 Transcription Factor Regulates Human ARTS Promoter Activity through Multiple Binding Sites
Source: PLoS One. 2015 Mar 19;10(3):e0120072. doi: 10.1371/journal.pone.0120072 (PMC4366172; doi:10.1371/journal.pone.0120072)
Supplement: S1 Table — All the primers used in this study are shown and the restriction sites or the mutated residues are underlined. (PDF) [file pone.0120072.s001.pdf]

Supplementary Table 1. Primers used in the present study

primer	orientation	Sequence (5′→ 3′)	Purpose
A1 F	Forward	CGAGCTCTGCCTTTTGAAATGTGTCTGTGTCC	Reporter
A2 F	Forward	CGAGCTCCTTTAGAGGTTCCCTTTCAG	Reporter(deletion)
A3 F	Forward	CGAGCTCGAAGGTTTCTCCTCAACCGG	Reporter(deletion)
A4 F	Forward	CGAGCTCGTCAGAGGAAGAGTGGCCCAG	Reporter(deletion)
A5 F	Forward	CGAGCTCTGCAGCCATGGTAAGGCA	Reporter(deletion)
R	Reverse	CCGCTCGAGTAGAGCGCAGGCATCACCCA	Reporter(deletion)
M1 F	Forward	AGGCAAGGCTACACCTACCTGTTAAAGCCACT	Reporter(mutation)
M1 R	Reverse	AGTGGCTTTAACAGGTAGGTGTAGCCTTGCCT	Reporter(mutation)
M2 F	Forward	AGGTACAGGCCCCTACCCGGGGCCGACTCGC	Reporter(mutation)
M2 R	Reverse	GCGAGTCGGCCCCGGGTAGGGGCCTGTACCT	Reporter(mutation)
Sp1(a) F	Forward	GTCAGAGGAAGAGTGGCCC	ChIP
Sp1(a) R	Reverse	ACAAATTATCCAGGGATCATC	ChIP
Sp1(b) F	Forward	TTGAATCCCTGCGGTAACTA	ChIP
Sp1(b) R	Reverse	ACTCAGCTCCAGAAGCACCGC	ChIP
Sp1(c) F	Forward	TGACTCTGCATACTCTGCGCATC	ChIP
Sp1(c) R	Reverse	AGTGCGGACCGCTGCGGGAG	ChIP
